# Clinical characteristics and molecular etiology of partial 17α-hydroxylase deficiency diagnosed in 46,XX patients

**DOI:** 10.3389/fendo.2022.978026

**Published:** 2022-12-15

**Authors:** Duoduo Zhang, Fengxia Yao, Min Luo, Yanfang Wang, Tiffany Tian, Shan Deng, Qinjie Tian

**Affiliations:** ^1^ Department of Obstetrics and Gynecology, Peking Union Medical College Hospital, Peking Union Medical College, Chinese Academy of Medical Sciences, Beijing, China; ^2^ Clinical Research Laboratory, Peking Union Medical College Hospital, Peking Union Medical College, Chinese Academy of Medical Sciences, Beijing, China; ^3^ Department of Biology, Emory College of Arts and Sciences, Atlanta, GA, United States; ^4^ Center for Rare Diseases Research, Chinese Academy of Medical Sciences, Beijing, China

**Keywords:** disorders of sex development, *CYP17A1*, partial 17α-hydroxylase deficiency (p17OHD), recurrent ovarian cyst, fertility preservation

## Abstract

**Introduction:**

Complete 17α-hydroxylase deficiency (17OHD) is relatively common, with typical juvenile female genitalia, severe hypertension, hypokalemia, and the absence of sexual development, but partial (or non-classical) 17OHD (p17OHD) is extremely rare. The p17OHD patients can present with a broad spectrum of symptoms in 46,XX karyotype including various degree of spontaneous breast development after puberty, recurrent ovarian cysts, oligomenorrhea and infertility depending on specific gene mutations and other influencing factors.

**Methods:**

This paper is a retrospective analysis of p17OHD cases from 1997 to 2021 in a Chinese tertiary hospital. Eight patients were recruited from unrelated families according to clinical data. Genotypes of patients were determined by sequencing the *CYP17A1* genes. Clinical characteristics were summarized based on manifestations, hormone profiles, and responses to treatments.

**Results:**

All seven post-pubertal patients had abnormal menses. All patients had enlarged multilocular ovaries, and six (6/8) had a history of ovarian cystectomy prior to a definite diagnosis of p17OHD. All eight patients’ sex hormone levels were in accord to hypogonadism with mildly elevated follicle-stimulating hormone levels, and oral contraceptives effectively suppressed the ovarian cysts. Of the four patients who underwent plasma renin activity tests, all showed results below the reference range. Fourteen alleles with a *CYP17A1* mutation were found. Exon 6 was the most frequent mutation site (5/14), and four out of these five mutations were c.985_987delTACinsAA, being the most common one. In Case 2, c.1220dupA was a newly reported mutation of *CYP17A1*.

**Conclusions:**

46,XX p17OHD patients were born with highly fragile ovarian reserve due to diverse mutations of *CYP17A1.* However, their multi-ovarian cysts can be managed conservatively for fertility preservation. This study focuses on p17OHD in 46,XX by locating the complex genetic causes in novel mutations, summarizing the puzzling spectrum of clinical manifestations, and illustrating the significance of fertility preservation in these scarce cases.

## Introduction

17α-hydroxylase/17,20-lyase deficiency (17OHD) is a rare condition that accounts for only 1% of congenital adrenal hyperplasia (CAH), occurring in 1 out of 1,000,000 births worldwide (1). Cytochrome P450 17α-hydroxylase (P450c17) is encoded by *CYP17A1*, which is located on chromosome 10q24–25 (2) and contains eight exons and seven introns (3). Similar to other CAH, 17OHD follows an autosomal recessive inheritance pattern and equally affects individuals harboring the 46,XY or 46,XX karyotypes (4). Depending on the retained degree of 17α-hydroxylase/17,20-lyase activities of P450c17, 17OHD can be either complete or partial.

Complete or classical 17OHD is relatively common and occurs when 17α-hydroxylase and 17,20-lyase activities are completely lost. As a result, complete 17OHD presents with an overproduction of mineralocorticoids, particularly 11-deoxycorticosterone (DOC), accompanied by an impairment in the production of glucocorticoids, androgens, and estrogens. Phenotypically, the disease is characterized by typical juvenile female genitalia, severe hypertension, hypokalemia, and the absence of secondary sex development (5).

Nonetheless, partial or non-classical 17OHD (p17OHD) is an extremely infrequent condition, characterized by isolated 17,20-lyase deficiency (ILD) and the partial loss of 17α-hydroxylase and 17,20-lyase function. The key difference between partial and complete17OHD is whether P450c17 exhibits, even a little, function. With some remaining P450c17 enzymic activity, clinical presentations of 46,XX patients with p17OHD include spontaneous menarche, irregular menses, oligomenorrhea or primary amenorrhea, spontaneous breast development, sparse or absent axillary and pubic hairs, juvenile genitalia, and the presence/absence of mild hypertension or hypokalemia. Laboratory results for p17OHD show increased plasma adrenocorticotropic hormone (ACTH) levels with (in ILD) or without (in p17OHD combined with 17,20-lyase deficiency) increased 17α-hydroxyprogesterone (17OHP) levels. Levels of follicle-stimulating hormone (FSH) and luteinizing hormone (LH) remain in the range of normal to mildly elevated, in contrast to the significantly elevated FSH (>40IU/L) in the complete type. Progesterone (P) levels were abnormally higher, while estradiol (E2) and testosterone (T) levels were lower when comparing to normal 46,XX female.

Herein, we retrospectively summarized eight clinically diagnosed 46,XX p17OHD patients in our department over the past 24 years and analyzed their clinical presentations, hormonal changes, genetic mutations, treatments, and prognosis. Because of 46,XX p17OHD patients’ atypical manifestations, they are often misdiagnosed and may receive inappropriate medical or operational treatments. To our knowledge, this is the first ever study to focus specifically on 46,XX p17OHD. We hope to clarify its genetic basis, differentiate its diagnosis from similar diseases, and guide the management of p17OHD in 46,XX, especially in treatments relevant to fertility.

## Materials and methods

### Patients and clinical data

Patients with p17OHD were included in this study if their clinical and biochemical manifestations were in line with p17OHD, if their peripheral blood karyotype was 46,XX, and if medical histories were completely documented. This study was approved by the Ethics Committee of Peking Union Medical College Hospital (IRB number: JS-2510). Written informed consent was obtained from the individuals ≥18-year-old and minors’ legal from guardian for patient < 18 year-old for the publication of any potentially identifiable images or data included in this article.

Medical data from 1997 to 2021, including age, gender, sexual intercourse history, chief complaints, diagnosis and treatment process, menstrual history, and family history, were retrospectively collected from medical records. Although genetic tests for *CYP17A1* mutations are crucial for the final diagnosis, initial diagnosis upon visit primarily depends on clinical manifestations and laboratory findings.

Physical examination and developmental status were recorded at the first visit. Breast and pubic hair development were assessed using the Tanner rating scale. Laboratory investigations included (1) gonadal function (FSH, LH, E2, P and T) and transvaginal/transabdominal sonography for the female reproductive system; and (2) ACTH, 17OHP, cortisol (F), urine free cortisol (UFC), dehydroepiandrosterone sulfate (DS), aldosterone (ALDO), plasma renin activity, serum potassium (K^+^), and CT for adrenal glands. The fasting blood sampling is taken at around 8 a.m. Since all postpubescent patients suffer from amenorrhea or oligomenorrhea, sexual hormones are measured on first visit at clinics without specific timing of period. Hormone tests were performed using an automated Elecsys immunoanalyzer (Beckman Coulter UniCel DXI800, Beckman Coulter; Brea, CA, USA). The follow-up time ranged from two months to more than 20 years.

### Mutation analysis of *CYP17A1* genes by Sanger sequencing

Blood samples were collected for sequencing of the *CYP17A1* gene (NM_000102.3). Genomic DNA from peripheral blood was extracted using the Lab-Aid 820 Automatic DNA Extraction Kit (Xiamen Zhishan Biotechnology Co., Ltd., China), according to the manufacturer’s instructions. Exons and intron-exon boundaries of *CYP17A1* were tested using Sanger sequencing.

Bioinformatic analyses were performed using an in-house pipeline. Briefly, sequencing reads that passed quality filtering were aligned with the human reference genome, hg19, using the Burrows–Wheeler Aligner program. Variants were identified using a combination of the FreeBayes, Genome Analysis Toolkit, CNVnator, and in-house software programs. All mutations were compared with databases such as dbSNP, ExAC, or 1000 Genomes Project, and the frequency of occurrence was calculated for different population groups. Predicted pathogenicity data were obtained using SNPEff, Ensembl Variant Effect Predictor, Sorting Intolerant from Tolerant, Polymorphism Phenotyping version 2, and dbscSNV databases. Gene-phenotype association was characterized from structured resources, including the online Mendelian Inheritance in Man database, and unstructured resources, including the literature, using natural language processing techniques. The average sequencing depth was >100×. Candidate variants identified in the exome data were validated using Sanger sequencing.

## Results

### General clinical characteristics

Eight patients were eligible for analysis (**Table 1**). All patients were presented as female with the 46,XX karyotype. Their first visits were between the ages of 11 and 36 years, and their initial presentation was primarily due to abnormal menses (7/8, 87.5%) or recurrent ovarian cysts before menarche (1/8, 12.5%). Except for one pediatric patient (Case 4), patients after puberty (>18 years) all exhibited spontaneous breast development. However, their breasts tended to be less well developed in relation to other peers of the same age (Tanner stages II-IV). None of the patients had well-developed axillary or pubic hairs. The external genitalia were underdeveloped, considering their ages, and showed juvenile patterns (hypoplastic labia majora and minora). Only Cases 3, 5, and 6 attempted to have sexual intercourses, but had unsatisfactory results due to the dryness of vagina.

**Table 1:** General characteristics of eight patients with partial 17α-hydroxylase deficiency.

Case	Age at first visit (years)	Chief complain	Height(cm)	Weight(kg)	Breast (Tanner)	Pubic /axillary hair (Tanner)	Juvenile female genitalia	Menstrual history	Blood pressure (mmHg)
1	19	Irregular menses	160	48	III	I	+	1~8 d/15~180d	140~160/ 110~120
2	18	Primary amenorrhea	169	54	II	I	+	N/A	120/80
3	27	Secondary amenorrhea for 13 years	158	61	IV	I	+	Secondary amenorrhea since 14yrs	110/60
4	11	Recurrent ovarian cysts	149	39	I	I	+	N/A	125/50
5	25	Primary amenorrhea	160	45	II	I	+	N/A	121/70
6	36	Oligomenorrhea for 6 years	160	51	V	I	+	4~7d/60~210d	130/80
7	20	Primary amenorrhea	164	49	II	I	+	N/A	90/60
8	21	Secondary amenorrhea for 3 years	179	65	III	I	+	Secondary amenorrhea since 18yrs	110/60

d, days; N/A, not available.

### Gonadal axis function

**Figure 1:**
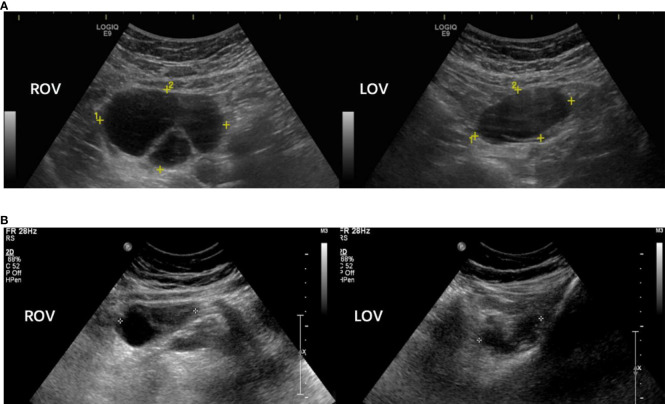
Sonographic pictures of a 18 year-old p17OHD patient’s multi-locular ovaries before **(A)** and after **(B)** combined oral contraceptive treatment (Case 2). Note: Rov, right ovary; Lov, left ovary.

The function of the hypothalamus-pituitary-ovary axis in our patients indicated hypogonadism (low serum E2 and T levels) with mildly to moderately elevated FSH and LH levels, despite that their serum P levels were abnormally high (16.7 ± 5.4 ng/mL) (**Table 2**). Each of the eight patients had ipsilateral or bilateral enlarged ovaries with a multilocular morphology (**Figure 1A**). Except for Cases 5 and 6, all other patients underwent ovarian cystectomy at least once, and their pathologies fit the diagnosis of luteinized cysts. Cases 2, 3 and 8 received combined oral contraceptives (COC) following surgery, and no relapse of ovarian cysts occurred (**Figure 1B**). Case 1 underwent oophorectomy after a persistent recurrence of multilocular cysts in both ovaries, even after COC treatment, and the cysts turned out to be serous cystadenoma. All patients’ ovarian tumor markers were within the normal reference range.

**Table 2:** Function of gonadal axis of eight patients with partial 17α-hydroxylase deficiency.

Case	FSH(IU/L)	LH (IU/L)	E_2_ (pg/mL)	P(ng/L)	T (ng/mL)	PRL (ng/mL)	Bone age	Tumor markers	Ovaries under ultrasonography
**1**	14.6	15.8	34.1	9.8	0.1	6.7	2-3 yrs delayed	CA_125_ 9.8U/ml	Left ovary: 5.6 cm×4.8cm, multilocular cysts Right ovary: 6.2 cm×4.2cm, multilocular cysts
**2**	18.4	26.5	18.5	23.6	<0.1	15.6	5-6 yrs delayed	CA_125_ 25~65U/ml	Left ovary: 5.9 cm×2.4cm, multilocular cysts Right ovary: 6.6 cm×5.4cm, multilocular cysts
**3**	9.4	18.2	15.8	36.8	<0.1	36.8	N/A	N/A	Both polycystic ovarian morphology with a 3.6cm cyst in the right ovary
**4**	11.2	22.7	17.6	17.9	<0.1	5.1	N/A	AFP 1.1 ng/ml,CEA 0.7 ng/ml,CA_199_ 6.2 U/ml,CA_125_ 14.5U/ml	Both polycystic ovarian morphology with a 5.0cm cyst in the right ovary
**5**	9.5	15.0	29.2	28.9	0.4	12.8	N/A	N/A	Left ovary: 7.2 cm×5.2cm, multilocular cysts Right ovary: 5.4 cm×3.0cm, multilocular cysts
**6**	4.3	12.5	19.9	8.5	1.7	7.9	N/A	AFP 2.19ng/ml,CA_125_10.74U/ml	Left ovary: 6.0 cm×3.5cm, multilocular cysts Right ovary: 5.5 cm×3.2cm, multilocular cysts
**7**	18.6	26.8	10.5	21.7	<0.1	5.7	3-5 yrs delayed	CA_125_ 27.15U/ml,AFP 1.18 ng/ml	Left ovary: 6.6 cm×6.0cm, multilocular cysts Right ovary: 4.8 cm×2.6cm, multilocular cysts
**8**	10.3	19.3	32.0	17.8	<0.1	14.0	N/A	CA_125_ 35U/ml,CA_199_ 4.9 U/ml,CEA 0.4 ng/ml,AFP 1.0ng/ml	Left ovary: nothing abnormal detected Right ovary: 10.3 cm×6.3cm, multilocular cysts, Doppler: resistance index 0.48

E2, estradiol; FSH, follicle stimulating hormone; LH, luteinizing hormone; PRL, prolactin; T, testosterone; yrs, years old.N/A, not available.

### Adrenal axis function

All patients had abnormally high ACTH. Cases 1, 2, 4, 5, and 8 had reduced serum F levels (**Table 3**). Apart from Cases 4, 5, and 8, the other five patients had increased serum 17OHP levels. Four patients (Cases 1, 2, 3, and 7) underwent the plasma renin activity test, and the results were remarkably below the lower reference. Low serum K^+^ levels were identified in Cases 1, 5, and 8. In addition, hypertension was observed only in Case 1 (**Table 1**). Computed tomography revealed enlarged adrenal glands in Cases 1, 2, and 4. No case received ACTH stimulation test.

**Table 3:** Function of adrenal axis of eight patients with partial 17α-hydroxylase deficiency.

Case	ACTH (pg/mL)	17OHP (ng/mL)	F (ug/dL)	DS (μg/dl)	UFC (μg/24h)	ALDO (ng/dL)	PRA (μg/L/h)	K^+^ (mmol/L)
REF	0~46.0	0.3~1.7	6.3~19.3	12~133	12.3~103.5	6.5~22	0.93~6.56	3.5-5.5
1	446.3	5.1	3.1	N/A	9.5	32.8	0	3.4
2	133.1	2.9	1.8	<50	<0.2	14.7	0.8	4.7
3	129.4	9.3	9.6	3.6	3.2	9.3	0	4.6
4	63.0	0.5	5.0	3.0	N/A	N/A	N/A	4.2
5	316.2	1.2	3.7	N/A	N/A	N/A	N/A	3.1
6	53.8	1.9	12.6	N/A	N/A	N/A	N/A	4.0
7	47.7	7.4	8.3	10.1	N/A	12.0	0.3	4.0
8	163.6	0.7	5.0	5.0	N/A	N/A	N/A	2.9

17OHP, 17α-hydroxy progesterone; 24h UFC, urine free cortisol; ACTH, adrenocorticotropic hormone; ALDO, aldosterone; DS, dehydroepiandrosterone sulfate; F, cortisol; PRA, plasma renin activity; REF, reference range.N/A, not available.

### Detection of genetic mutations

**Figure 2:**
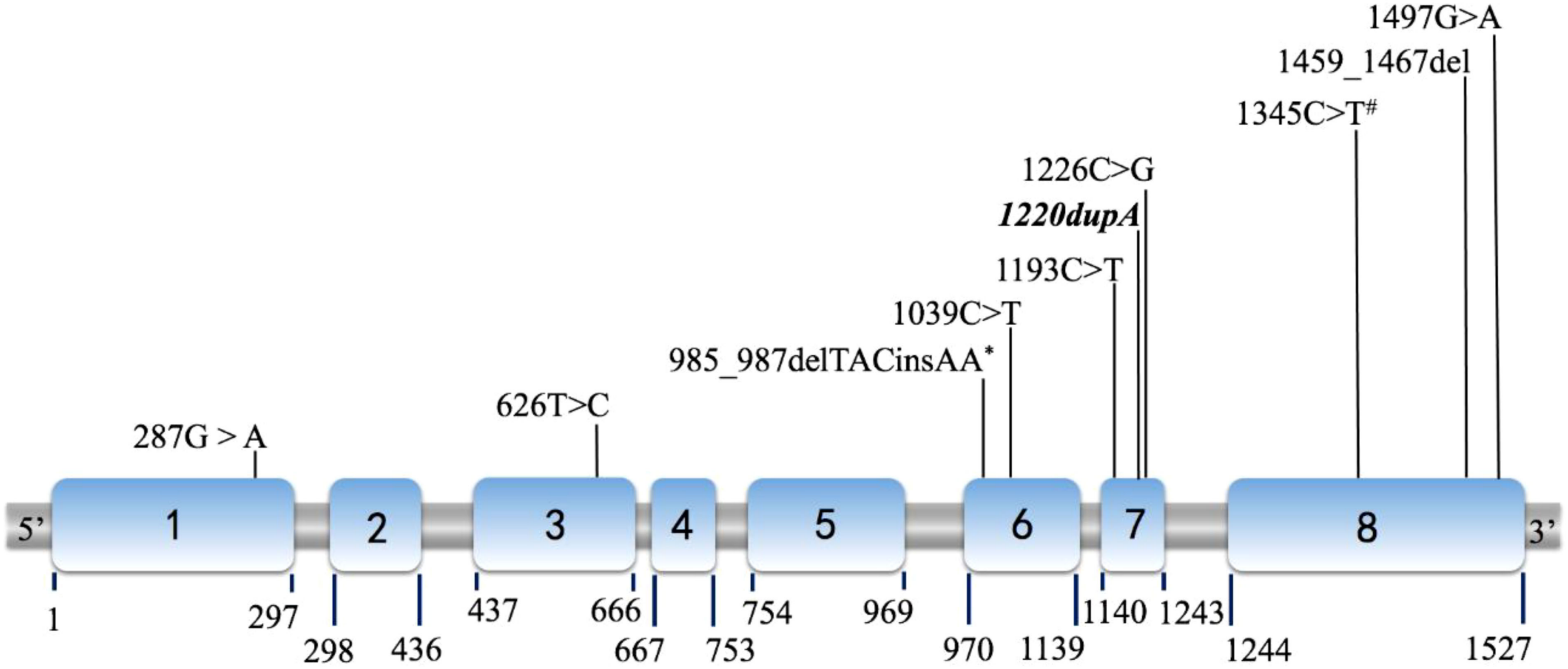
Diagram of the P450c17 cDNA (NM_000102) with location of mutations. The locations shown as Italic and Bold indicate novel mutations (1220dupA in case 2). Among all 14 mutations detected, mutations marked by asterisk (*) were discovered in 4 cases (Case 1, 3, 4 and 8); and mutations marked by hashtag (#) were found in both case 3 and 5. Other 8 mutations were identified as unique for each case respectively.

The detected gene mutations are summarized in **Table 4** and **Figure 2**. Among the eight patients clinically diagnosed with p17OHD, *CYP17A1* gene mutations were detected in all patients but only 14 alleles mutations (6*2+2*1) were identified. Six patients had two heterozygous allele mutations, and two patients had only one single allele mutation (Cases 6 and 8). All mutations were heterozygous among those with two known mutant alleles. Exon 6 (5/14) was the most frequent mutation site, and four out of these five mutations were c.985_987delTACinsAA (p.Tyr329LysfsTer90), which was the most common mutation pattern. The c.1220dupA of *CYP17A1* from Case 2 was a newly reported mutation. According to the 2015 American College of Medical Genetics and Genomics (ACMG) guideline, this new variant was defined as a variant of pathogenic (PVS1+PS2).

**Table 4:** Summary of gene mutations in eight patients with partial 17α-hydroxylase deficiency.

Case	Gene	Base change	Amino acid change	Location	Type of mutation	dbSNP	Scores of SIFT prediction	Prediction(cutoff=0.05)	ACMG guideline
1	*CYP17A1*	c.985_987delTACinsAA	p.Tyr329LysfsTer90	Exon 6	Frameshift	N/A	–	LOF	pathogenic
	*CYP17A1*	c.1193C>T	p.Ala398Val	Exon 7	Missense	rs1315561755	0.00	Deleterious	pathogenic
2	*CYP17A1*	c.1220dupA*	p.His407GlnfsTer8	Exon 7	Frameshift	N/A	–	LOF	pathogenic
	*CYP17A1*	c.1497G>A	p.Trp499Ter	Exon 8	Nonsense	rs776625979	–	LOF	pathogenic
3	*CYP17A1*	c.985_987delTACinsAA	p.Tyr329LysfsTer90	Exon 6	Frameshift	N/A	–	LOF	pathogenic
	*CYP17A1*	c.1345C>T	p.Arg449Cys	Exon 8	Missense	rs371825363	0.00	Deleterious	pathogenic
4	*CYP17A1*	c.985_987delTACinsAA	p.Tyr329LysfsTer90	Exon 6	Frameshift	N/A	–	LOF	pathogenic
	*CYP17A1*	c.1039C>T	p.Arg347Cys	Exon 6	Missense	rs104894149	0.00	Deleterious	pathogenic
5	*CYP17A1*	c.1226C>G	p.Pro409Arg	Exon 7	Missense	rs367833709	0.00	Deleterious	pathogenic
	*CYP17A1*	c.1459_1467del	p.Asp487_Phe489del	Exon 8	Deletion	rs756135168	0.00	Deleterious	pathogenic
6	*CYP17A1*	c.626T>C	p.Leu 209 Pro	Exon 3	Missense	N/A	0.00	Deleterious	pathogenic
	N/D	N/D	–	–	–	–	–	–	–
7	*CYP17A1*	c.287G>A	p.Arg96Gln	Exon 1	Missense	rs104894153	0.00	Deleterious	pathogenic
	*CYP17A1*	c.1345C>T	p.Arg449Cys	Exon 8	Missense	rs371825363	0.00	Deleterious	pathogenic
8	*CYP17A1*	c.985_987delTACinsAA	p.Tyr329LysfsTer90	Exon 6	Frameshift	N/A	–	LOF	pathogenic
	N/D	N/D	–	–	–	–	–	–	–

ACMG, American College of Medical Genetics and Genomics, dbSNP, single nucleotide polymorphism database; LOF, loss of function; N/A, not available; N/D, not detected; rs, reference SNP; SFIT, sorts intolerant from tolerant.

*a novel mutation.

## Discussion

In p17OHD, P450c17, with some remaining function, maintains certain activity in estrogen synthesis that result in various degrees of female secondary sexual characteristics development and mild symptoms of mineralocorticoid hyperactivity [hypertension, 12.5% (1/8); hypokalemia, 37.5% (3/8)] due to the limited overproduction of DOC. Therefore, all post-pubertal cases exhibited various extents of spontaneous breast development, although their breasts tended to be less developed in relation to other peers of the same age, due to the impaired and limited activity of P450c17. In 46,XX patients, p17OHD is atypical in endocrinology but the recurrent multiple ovarian cysts distinguish p17OHD from the more common complete 17OHD (6). Therefore, summarizing the characteristics of p17OHD is of great clinical significance.

### Mutations in patients with p17OHD

Human *CYP17A1* is located on chromosome 10q24.3, and spans 6.6 kb and eight exons (7). According to previous studies, the c.985_987delTACinsAA frameshift mutation is the most prevalent *CYP17A1* mutation among Chinese patients, and is present in approximately 50% of patients with complete 17OHD (8). Due to the founder’s effect in Asia, this mutation has been reported mainly in China and Korea (9). Similarly, the c.1459_1467del mutation found in Case 5 is a deletion of GACTCTTTC commonly seen in China and Thailand (10). Mutations in exons 6 and 8 are the most prevalent mutations among Chinese patients with 17α-hydroxylase/17,20-lyase deficiency (11).

In our series, four patients had mutations in exon 6, four in exon 8, and three in exon 7. The carboxyl (C) terminus of P450c17 is fundamental for heme (an electron carrier) binding, so even the slightest C terminal alternation can destroy almost all enzyme activities (12). For example, according to the computed model of human P450c17, p.Arg347Cys caused by c.1039C>T (Case 4) results in a charged aspartate, weakening the hydrophobic interaction and therefore destabilizing the entire enzyme structure (12). The mutation is located in the redox-partner interaction domain that contains key residues involved in the interaction with redox proteins and contributes to the positive charges on the proximal surface of P450c17. Geller et al. reported that the absence of charged amino acids in that domain selectively impaired 17,20-lyase activity without substantially reducing 17α-hydroxylase activity (13). Mutations p.Arg347His or Cys and p.Arg358Gln map to the P450c17 surface, which impair the electrostatic interactions of P450c17 with POR and CYB5A (14), explaining the selective ILD observed. An *in vitro* study showed that the p.Arg347Cys mutant protein had 13.6% and <1% residual activities of 17α-hydroxylase and 17,20-lyase, respectively (15). Residue Ala398 comprises part of the C terminus of the K helix, a motif present in all known cytochrome P450 systems (16). The structural importance of this motif corresponds to the detrimental effect of p.Ala398Val (c.1193C>T, Case 1). In addition, the novel mutation p.His407GlnfsTer8 (c.1220dupA, Case 2) seems to have a similarly damaging effect, because of its identical location in the K helix. Both p.Pro409Arg (c.1226C>G, Case 5) and p.Arg449Cys (c.1345C>T, Cases 3 and 7) lay in a region critical for heme binding within a contiguous three-dimensional space (12).

Only one mutant allele was detected in Cases 6 and 8, which is paradoxical to the etiology of an autosomal recessive disease. Both patients had mild manifestations, particularly mineralocorticoid symptoms (**Table 3**). This could be due to large fragment deletions of the *CYP17A1*. Nevertheless, introns also influence *CYP17A1* expression. In this study, we sequenced the exons and flanking intronic DNA. Mutations in the un-sequenced intron area may affect the splicing process, making the spliceosome unable to properly remove introns and join both exon ends of the *CYP17A1* mRNA (17). The precise identification of the splice site is demanding, because the consensus sequences are very brief and there are many other sequences similar to the consensus motifs of the classical splice sites. Further analysis of *CYP17A1* introns may provide a deeper understanding of the regulation and expression of *CYP17A1*.

### Pathophysiology of p17OHD and related functional ovarian cysts in 46,XX p17OHD

Although all eight patients had ACTH above 46 pg/mL and five had F below 6.3 μg/dL (cases 1, 2, 4, 5 and 8), none of them received glucocorticoid replacement after receiving correct diagnosis and before the consideration of fertility treatment. Glucocorticoid therapy induces adrenal axis suppression and possible failure to augment appropriate corticosterone production during acute stress (18). Since DOC accumulates upstream of the block by the failed enzyme, this mineralocorticoid causes low-renin hypertension, although it is slightly less potent than ALDO (4). The amount and activity of DOC are not sufficient to saturate the mineralocorticoid receptor under p17OHD conditions. This is consistent with our seven patients (case 2 to 8), none of whom had symptoms of glucocorticoid deficiency or an excess of mineralocorticoid. All four patients that underwent plasma renin activity tests showed results below the reference range, but only the ALDO of case 1 is dramatically elevated, which accords with her hypertension and hypokalemia.

Pathologically and persistently elevated P level is a basic indicator in most types of CAH, while the 17OHP and DHEA are substantial intermediate products in the steroid metabolism (5). The variation of them often indicates the non-classical type of a particular enzymatic deficiency in CAH. For example, in patients with p17OHD due to the combined defect of the partial 17-hydroxylase and 17,20-layse, we can observe an increased P and 17OHP, but decreased DHEA. For patients with ILD, the elevation of 17OHP and decreased DHEA can be more obvious than those with 17-hydroxylase dysfunction alone. Due to the distinct allele mutation and variant combination of both mutated alleles, the remaining of enzymic function varies dramatically. Therefore, the specific value of a particular biochemical parameter is not practical in the determination of p17OHD, but they can be helpful for clinical suspicion of a certain non-classical type in CAH.

A signature symptom in p17OHD patients with the 46,XX karyotype is recurrent ovarian cysts (19). In our study, recurrent ovarian cysts occurred in all patients, and 75% (6/8) had ovarian cystectomy at least once. Furthermore, three cases had undergone two or more invasive ovarian operations, where the youngest patient (Case 4) was only 10 years old at the time of surgery. Most patients underwent unnecessary ovarian operations without realizing the presence of p17OHD. The mechanism underlying recurrent ovarian cysts might be related to persistent stimulation of raised gonadotropins (FSH and LH) on residual ovarian follicles. But the compromised secretive function of follicles in p17OHD patients leads to low level of estrogen and weak negative pituitary feedback. Such phenomenon is rarely seen in complete 17OHD, because there are no viable follicles in the complete form due to extremely severe enzymatic defects. As a result, no follicle genesis or response to the high levels of gonadotropins exists.

The COCs provide an effective conservative management for the above type of recurrent cysts. Large cysts shrink soon after COC administration in most cases through suppressing the gonadotropin stimulation from hypophysis, but ovarian cysts may reappear after the cessation of COC treatment and it is still effective to use COC again to repress the cyst. However, if ovarian cysts persist after COC intervention, the possibility of non-functional tumors (e.g., Case 1, serous cystic adenoma) should be excluded.

### Fertility in 46,XX patients with p17OHD

Fertility is an important issue in patients with p17OHD and their families. Almost all patients of reproductive age in this study had primary infertility. Currently, it is impossible for patients with complete 17OHD to be fertile spontaneously because of the premature exhaustion of primordial follicles (20). For p17OHD, patients have reduced fertility due to the insufficiency of E2 and T synthesis, and asynchronism of endometrium by chronic P excess, and there have been no reports of spontaneous natural pregnancy in patients with the 46,XX p17OHD. Fortunately, with the development of assisted reproductive technology (ART), pregnancy can be achieved by decreasing the high P with corticosteroids and creating an artificial menstrual cycle for *in vitro* fertilization and embryo transfer (21). During a successfully controlled ovarian stimulation, E2 remains relatively low compared to the number of growing follicles, but the low E2 may not affect viable embryo development (22). Yet, the persistent abnormally high P will interfere with the simultaneous development of the fertilized egg and endometrium (23, 24). Therefore, successful artificial endometrial preparation can be achieved with glucocorticoid supplementation and conventional hormone replacement in frozen-thaw cycles. Consequently, pregnancy can be achieved with ART if 46,XX p17OHD are correctly diagnosed and ovaries are well protected by carefully avoiding unnecessarily repeated cystectomy or even oophorectomy, as cysts spontaneously disappear following the commencement of COC (25). Doing so is essential for protecting the diminished ovarian reserve and saving the chance for future ART in 46,XX p17OHD patients.

## Limitations

This study has several limitations. Primarily, as p17OHD is a very rare type of CAH, the sample size is very limited; however, we will continue to collect and update data. Secondly, some laboratory works can be modified if without consideration of cost [e.g., mass spectrometry for steroids applied in all patients and assessment of adrenal function by serum F before and after ACTH stimulation, which is the accepted standard for CAH diagnosis (18)]. Last but not the least, the functional changes of the genetic mutations we found have not been examined and confirmed *in vitro* or *in vivo*, and their family pedigrees can be investigated.

## Conclusion

The p17OHD patients are left with different residual activity of P450c17, and varied function in the remaining follicles in 46,XX female patients contribute to distinct degrees of female secondary sexual characteristic development. Recurrent ovarian cysts are commonly occurring in these patients while not observed in the complete form of 17OHD. These cysts can be effectively suppressed using COCs instead of repeated ovarian cystectomy. Early diagnosis, timely treatments, and ART may make it possible for 46,XX p17OHD patients to retain fertility and have their own biological children. The mutant alleles in these patients were more likely to be heterozygous and we discovered that c.1220dupA was a novel detrimental mutation leading to frameshift mutations of *CYP17A1*.

## Data availability statement

The raw data supporting the conclusions of this article will be made available by the authors, without undue reservation.

## Ethics statement

The studies involving human participants were reviewed and approved by the Ethics Committee of Peking Union Medical College Hospital (IRB number: JS-2510). The patients/ participants or legal guardians of the children provided their written informed consent to participate in this study. Written informed consent was obtained from the individuals ≥18-year-old AND minors’ legal guardian for patients < 18-year-old for the publication of any potentially identifiable images or data included in this article.

## Author contributions

Conceptualization: QT and FY. Data curation: FY, YW and TT. Data collection: QT, SD, ML. Funding acquisition: QT. Investigation: FY and TT. Methodology: FY and DZ. Writing – original draft: DZ. Writing – review & editing: QT and FY. All authors contributed to the article and approved the submitted version.
